# Self-reported maternal morbidity: Results from the community level interventions for pre-eclampsia (CLIP) baseline survey in Sindh, Pakistan

**DOI:** 10.1016/j.preghy.2019.05.016

**Published:** 2019-07

**Authors:** Sana Sheikh, Rahat Najam Qureshi, Farrukh Raza, Javed Memon, Imran Ahmed, Marianne Vidler, Beth A Payne, Tang Lee, Diane Sawchuck, Laura Magee, Peter von Dadelszen, Zulfiqar Bhutta, the CLIP Working Group

**Affiliations:** a Division of Women & Child Health, Department of Obstetrics and Gynaecology, 2^nd^ Floor Private Wing, Aga Khan University, Stadium Road, Karachi 74800, Pakistan; b Department of Obstetrics and Gynaecology, University of British Columbia, 2329 West Mall, Vancouver, BC V6T 1Z4, Canada; c Centre for International Child Health, BC Children’s Hospital Research Institute, 950 W 28th Ave, Vancouver, BC V5Z 4H4, Canada; d Vancouver Island Health Authority, 2334 Trent Street, Suite 643 Victoria, Canada; e Department of Women and Children’s Health, School of Life Course Sciences, King’s College London, Strand, London WC2R 2LS, UK; f Program for Global Pediatric Research, Hospital for Sick Children, 555 University Ave, Toronto, ON M5G 1X8, Canada

**Keywords:** CLIP, Community Level Intervention for Pre-eclampsia, LMIC, Low- and middle-income countries, MMR, Maternal mortality ratio, MoH, Ministry of Health, HMIS, Health Management Information System, DHIS, District Health Information System, PDHS, The Pakistan Demographic and Health Survey, MDGs, Millennium Development Goals, NMR, Neonatal mortality ratio, Ucs, Union Councils, MWRA, Married women of reproductive age, IQR, Interquartile ranges, SPSS, Statistical Package for Social Sciences, WASH, Water, sanitation and hygiene, PCA, Principal Component Analysis, CBR, Crude birth rate, TBA, Traditional birth attendants, IMR, Infant mortality ratio, U5MR, Under five mortality ratio, SES, Socio-economic status, Community, Maternal newborn and child, Self-reported, Morbidity, Health estimates

## Abstract

**Objective:**

Community-based data regarding maternal and perinatal morbidity and mortality are scarce in less-developed countries. The aim of the study was to collect representative community-level demographic health information to provide socio-demographic and health outcome data.

**Methods:**

A retrospective household survey of women of reproductive age (15–49 years) living in two districts of Sindh Province, Pakistan was conducted. Pregnancy incidence over the past 12 months and during each woman’s lifetime; maternal, fetal, infant and child deaths in the past 12 months; and rates of hypertension and seizures in pregnancy were calculated.

**Results:**

From June to September 2013, 88,410 households were surveyed with 1.2 (±0.6) women of reproductive age per household. 19,584 women (11.9%) reported pregnancies in the preceding 12 months; 83.0% had live births, 3.5% resulting in stillbirths and 13.6% in miscarriages. 34.2% of deliveries occurred at home. Out of all women who reported a pregnancy in past 12 months, 62.1% reported high blood pressure and 11.9% reported seizures complicating her most recent pregnancy. Blood pressure was not measured during survey to confirm hypertension. The perinatal, neonatal and maternal mortality ratios were 64.7/1000, 39/1000 and 166/100,000 livebirths, respectively.

**Conclusion:**

This study estimated population-level mortality ratios that can be used for the planning of health interventions in these regions. Self-reported pregnancy hypertension and seizures was inaccurate, reflecting limited community understanding of these disorders. Mortality estimates are comparable to those reported by the World Health Organization for maternal mortality ratio and neonatal mortality rate of 170/100,000 and 36/1000 live births, respectively.

## Highlights

• Community-based estimates of maternal/perinatal death and morbidity are reported.
• Stillbirth led to increased self-report of hypertensive complications in the index pregnancy.
• Self-reported seizure and pregnancy hypertension is prone to error in regions of low literacy.

## Graphical abstract

**Figure:**
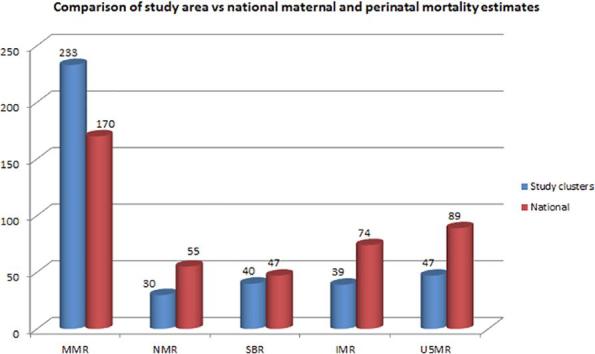

## 1 Introduction

Reliable data regarding maternal, newborn and child health are scarce in low- and middle-income countries (LMIC). Unfortunately, many countries with a high burden of maternal morbidity and mortality do not have reliable vital statistics systems [1]. Approximately 33% of the world’s annual births and 66% of the annual deaths are not documented in any vital registration database, particularly in Asia and Africa [1].

Pakistan has an inefficient civil registration system and a high estimated maternal mortality ratio (MMR) (170/100,000 live births) [2]. As the last national census in Pakistan was conducted in 1998, reliable estimates of population size and demographics are lacking. In the early 90s the Ministry of Health (MoH) established a Health Management Information System (HMIS) to inform planning, management and monitoring of health facilities to improve service delivery [3]. In 2007 this system was modified as the District Health Information System (DHIS) with wider scope and a revision of data collection indicators, tools and software. The aim of DHIS was to collect precise and timely data from health facilities to district health offices to set health priorities and allocate resource [4]. But exploration of use of HMIS/DHIS revealed that data quality was poor and was not efficiently used for decision-making or dissemination [3], [5], [6].

The Pakistan Demographic and Health Survey (PDHS) was conducted in 2012. Estimates of maternal mortality and morbidity are missing in this report [7]. As a signatory of Millennium Development Goals (MDGs) maternal and child health is a priority issue for Pakistan’s government and efforts have been directed towards improving these indicators. The absence of timely data and effective monitoring systems preclude the estimation of the true health burden. Different national surveys report varying estimates for maternal and neonatal deaths (MMR 170–270/100,000 and neonatal mortality rate [NMR] 36–55/1000 live births) [2], [8], [9].

Reliable rates of maternal morbidity are particularly scarce, as available data are primarily derived from facility-based studies [10], [11], [12], [13]. The disease burden in health facilities may differ from the community burden. Health facility data may either over- or underestimate the burden of disease depending upon hospital- and population-level factors [14]. There are no reliable prevalence rates for pre-eclampsia, eclampsia, gestational diabetes mellitus and other pregnancy complications in Pakistan.

Health statistics are crucial for developing effective national and international health policies to improve maternal and child health. In the absence of an efficient vital events registration system, health indicators can be best gathered through household surveys [1]. The aims of this study were to collect community-level demographic health information to provide reliable socio-demographic and health outcome data. In addition, this study explored factors associated with self-reporting of hypertension and seizures in pregnancy.

## 2 Description of study setting

**Fig. 1:**
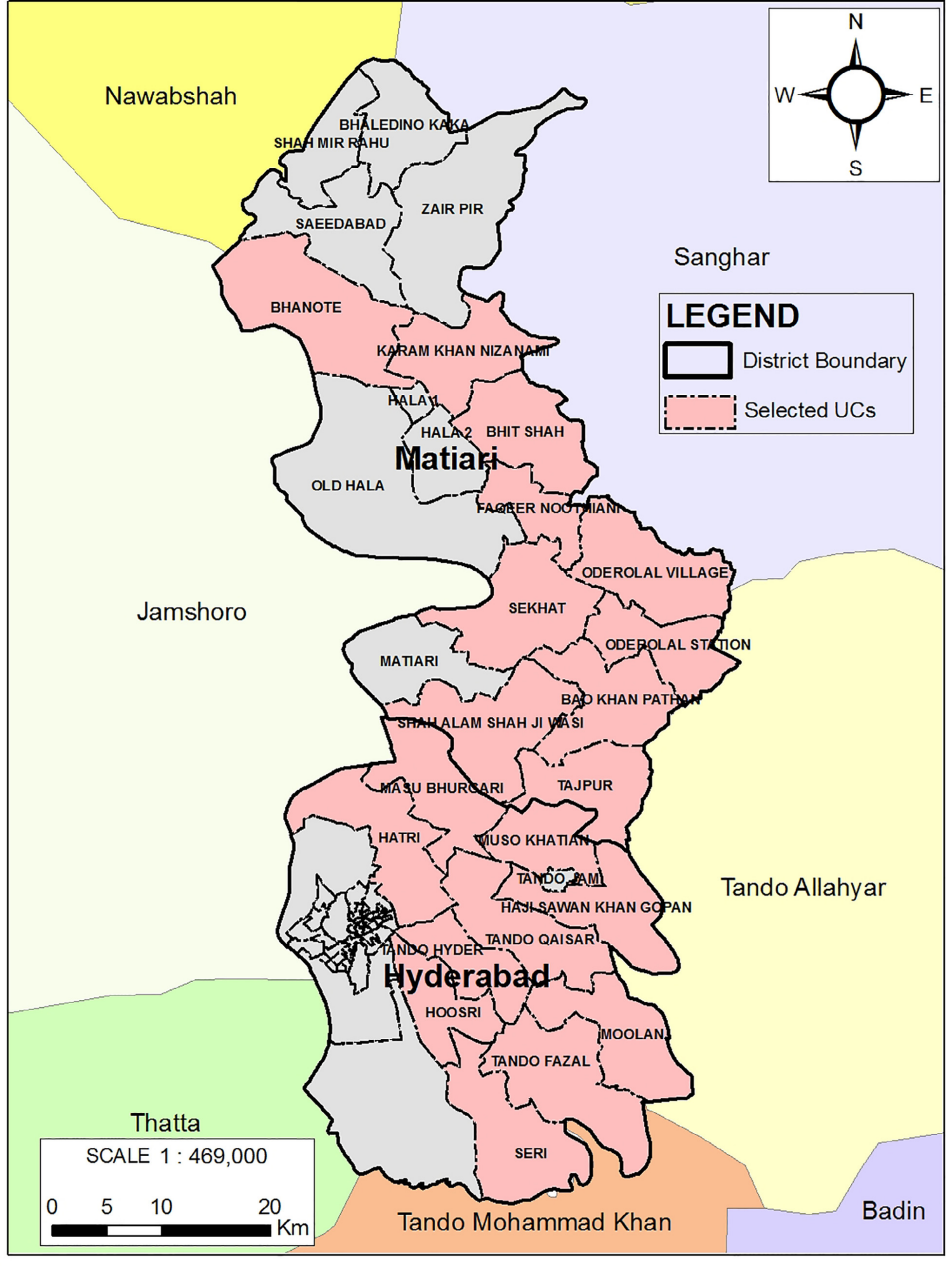
Map of study area.

This survey was conducted in twenty Union Councils (UCs) of Matiari and Hyderabad Districts of Sindh Province, Pakistan (Fig. 1.). Hyderabad has an estimated population of 4.5 million of whom 60% live in urban areas. It is the second-most urbanized district of Sindh after Karachi. Hyderabad is further divided into four sub-districts, Hyderabad city, Hyderabad rural, Latifabad and Qasimabad [15]. Matiari district is divided into three sub-districts, Matiari, Hala and Saeedabad [14]. The household survey was conducted in Hyderabad rural, Hala and Matiari sub-districts. The Hala and Matiari sub-districts are located 250 km north of Karachi, with an estimated population of 0.6 million. The area is largely agricultural and development indicators are representative of rural Sindh [16].

## 3 Material and methods

A cross-sectional household survey was conducted in twenty UCs of Matiari and Hyderabad districts, from June to September 2013. The survey was undertaken to ascertain baseline health data, and to identify women of childbearing age and those pregnant at the time of the survey. Every household in the study area was approached. In addition to household-level data, individual-level demographics and health information was collected on all married women of reproductive age (15–49 years). Due to cultural sensitivities and the stigma associated with pregnancies outside of marriage, the study population was limited to married women only. Health information was focused on current, recent (within the past 12 months) and past obstetric history.

Prior to the initiation of data collection permissions were obtained from community gate keepers for the study. Community leaders such as landlords, religious leaders, social activists or school teachers were considered as community gate keepers. In the region, a combined or extended family system is the preferred household structure; hence one house may include multiple families. Data for all eligible women in the same house were collected. A household was defined as the people sharing the same stove or kitchen. Verbal consent was obtained from the head of each household at the time of the survey. A structured questionnaire was administered by trained research staff to the available head of household for demographic information. Information regarding maternal, perinatal and child outcomes were obtained from all married women of reproductive age (MWRA). The questionnaire was translated to and administered in Sindhi, which is the local language of the study area.

This study received ethical approval from Ethics Review Committee, Aga Khan University, Karachi, Pakistan (ERC#1917-Obs-ERC-11); National Bioethics Committee of Pakistan (4-87/NBC-104/12/RDC/1895) and the Clinical Research Ethics Board, University of British Columbia, Vancouver, Canada.

Data were entered into an online database (RedCap) housed in Pakistan, and analyzed using Statistical Package for Social Sciences (SPSS) version 19.0 (IBM Corporation, Armonk, NY, USA). For descriptive analysis, means and standard deviations or median and interquartile ranges (IQR) depending on distribution of data for continuous variables and frequency and proportions for categorical variables were estimated. Factors associated with self-reported hypertension or seizures during pregnancy were explored through both univariate and multiple logistic regression to calculate crude and adjusted odd ratios. Adjustment factors considered were age of the women, socioeconomic status, type of birth attendant and place of birth. No statistical method was used to impute missing data.

## 4 Results

### 4.1 Socio-demographic indicators

The total population surveyed was 595,408 individuals in 88,410 households. The average household size was 6.7 ± 3.7 people. The total number of married women was 122,370, of whom 100,005 ((81.7%) were of reproductive age (15–49 years). The mean age of MWRA was 27.5 ± 4.4 years, and there were an average 1.2 ± 0.6 MWRA and 2.7 ± 2.3 children under 13 per household.

Nearly three quarters (70%) of heads of household were male; usually the eldest male household member. Sixty-three percent of male heads of household had no education, and were most commonly unskilled manual laborers (40%) and agricultural workers (34%). Among the female heads of household, 97.1% had no education.

Data collected for water, sanitation and hygiene (WASH) indicators showed that 99% of households have access to ‘improved drinking water’ [9]. The most common source of improved drinking water (73%) was a hand pump. Thirty-seven percent of households had ‘improved sanitation facilities’ [9]. Approximately two-fifths (43%) of the households had no toilet facility and were using the open bush or field. When hygiene practices were explored, it was found that 95% of people washed their hands after defaecation, and 60% of households had soap at the hand-washing place. Information on household characteristics revealed that 41% of houses had walls made of bricks or cement, 55% had roofs made of tiles, and 67% had sand or mud floors. (Table 1)

**Table 1:** Household demographics.

Variables	Total
	N = 88,410
	Frequency (%)
	Mean ± SD
	Median (IQR)
Total population	595,408
Married women	122,370 (20.5%)
Married women of reproductive age (15–49 years)	100,005 (16.7%)
Average number of children under 13 per household	2.7 ± 2.3
Average number of children under 5 per household	1.2 ± 1.2
Number of children 5–13 year old in a household attending school	0 (0, 1)
Households led by men	61,887 (70%)
Average years of school completed for male heads of household	8.1 ± 3.6
Illiterate male heads of household	55,761 (63.1%)

*Household access to improved drinking water:*	
Piped water	6888 (7.8%)
Tube well or borehole	15,470 (17.5%)
Hand pump	64,560 (73%)
Other	298 (0.3%)

*Household access to improved sanitation:*	
Flush or pour-flush to:	
-piped sewer system	31,842(36%)
-septic tank	254 (0.3%)
-pit latrine	12,098 (13.7%)
Ventilated improved pit latrine	135 (0.2%)
Pit latrine with slab	362 (0.4%)
Composting toilet	397 (0.4%)
Flooring material:	59,335 (67.1%)

*Mud or sand*	
Cement	26,702 (30.2%)
Tiles	1859 (2.1%)
Other	513 (0.6%)

*Wall material:*	
Mud	23,943 (27.1%)
Bamboo or sticks	5067 (5.7%)
Bricks or cement	36,173 (40.9%)
Bricks unplastered	22,219 (25.1%)
Other	1004 (1.1%)

*Roof material:*	
Thatch or palm leaf	34,584 (39.1%)
Tin sheets	816 (0.9%)
Roofing tiles	48,481 (54.8%)
Other	4525 (5.1%)

*Wealth quintiles:*	
Poorest + poor	35,364 (40%)
Middle	17,684 (20%)
Rich + Richest	35,362 (40%)

Socio-economic quintiles were estimated using a Principal Component Analysis (PCA)-based approach [17]. Forty percent of the study population was in the poorest and poor quintiles and this subgroup had 40% of the dependent population in the study (i.e. children under 13 years of age).

### 4.2 Pregnancy outcomes

Nineteen percent (19,584) of MWRA reported a completed pregnancy in the previous 12 months; while 12% (11,946) were pregnant at the time of survey. The estimated crude birth rate (CBR) was 28.4 per 1000 population and miscarriage rate of 13.6% out of all pregnancies completed in last 12 months. Of 16,946 births reported during the preceding 12 months, there were 16,261 livebirths and 685 stillbirths (40/1000 livebirths). Similar proportions of births occurred at private hospitals (35%) and at home (34%). Skilled birth attendant were present at 66.7% of home deliveries, while 32% of births were attended by traditional birth attendants (TBA) (Table 2). With respect to complications, 72% reported severe headache, 62.1% reported high blood pressure, and 11.9% reported seizures in their most recent pregnancy.

**Table 2:** Pregnancy outcomes.

Variables	Total
	N = 100,005
	Frequency (%)
	Mean ± SD
Married women of reproductive age with one or more pregnancy in the last 12 months	19,584 (19%)
Married women of reproductive age currently pregnant	11,946 (11.9%)
Pregnancy outcomes:	(n = 19,584)
Live births	16,261 (83%)
Stillbirths	685 (3.5%)
Miscarriages	2655 (13.6%)

*Deliveries conducted by:*	
Private doctor	5771 (34.1%)
Govt. doctor	4991 (29.5%)
Lady Health Visitor or midwife	535 (3.2%)
Dai or TBA	5496 (32.5%)
Other	137 (0.8%)

*Place of delivery:*	
Home	5798 (34.2%)
Govt. hospital	5104 (30.1%)
Private hospital	5965 (35.2%)
En route to facility	62 (0.4%)

*Self-reported complications:*	
High blood pressure	11,980 (61.2%)
Seizures	2325 (11.9%)
Severe headache	14,161 (72.3%)

*High blood pressure during pregnancy reported by:*	
Women herself	10,493 (62.1%)
Family member	1487 (55.5%)

Delivery and related information is missing for n = 2725 (13.9%) women, for high blood pressure n = 648(3.3%) reported ‘Don’t know’, for seizures n = 316 (1.7%) reported ‘Don’t know’ and n = 193(1%) were missing.

### 4.3 Mortality rates

In addition to the stillbirths noted above, there were 271 deaths of MWRA, including pregnancy-related and non-pregnancy-related deaths. (166/100,000 livebirths). The median maternal age at death was 40 years (IQR 30, 45). Four hundred and eighty-five deaths were reported in children less than one month of age and 275 in children between one month and five years. The estimated infant mortality rate (IMR) was 39/1000 livebirths and under five mortality rate (U5MR) was 47/1000 live births. More than half (55%) of neonatal deaths and 71% of deaths in children from one month to five years occurred at home. (Table 3)

**Table 3:** Mortality rates.

Variables	Total
	Frequency (%)
	Mean ± SD
	Median (IQR)
Deaths of married women of reproductive age	271
Age at death of married women of reproductive age (years)	40 (30, 45)
Neonatal deaths	N = 485
- Early neonatal deaths (0–7 days)	367 (75.7%)
- Late neonatal deaths (8–28 days)	118 (24.3%)
Child deaths (1 month to 5 years of age)	N = 275
- Infant deaths (1–12 months)	157 (57.1%)
- Child deaths (12–59 months)	118 (42.9%)
Infant mortality rate (per 1000 live births)	39
Under five mortality rate (per 1000 live births)	47
Stillbirth rate (per 1000 births)	40

*Place of neonatal deaths (0–28 days)*	
Home	266 (54.8%)
Other	219 (45.2%)

*Place of infant and child deaths (1–59 months)*	
Home	196 (71.3%)
Other	79 (28.7%)

### 4.4 Factors associated with self-reporting of complications in pregnancy

Seventy-two percent of women with a pregnancy in the preceding twelve months reported severe headache, 62.1% reported high blood pressure, and 12% reported seizures. Age and socio-economic status (SES) were associated with self-reported pregnancy hypertension and seizures (p-value < 0.05) at the univariate level. The highest and lowest two categories of SES were combined to explore the association between SES and self-reported complications. Women reporting complications were more likely to belong to a middle socio-economic class (mid-socio-economic class) (pregnancy hypertension OR 1.11 (95% CI 1.02, 1.21); seizures 1.39 (95% CI 1.24, 1.57)) compared with those without reported complications. The level of education of the male and female head of household was not associated with self-reporting complications for women of that household (p = 0.45).

There was a strong association between adverse pregnancy outcomes (miscarriage or stillbirth) and self-reported hypertension and seizures. The odds of having a miscarriage (gestational age up to 24 weeks) was 1.57 times (95% CI 1.43, 1.73) and stillbirth was 1.38 times greater (95% CI 1.16, 1.64) in women reporting hypertension in their most recent pregnancy compared to women with no complications reported. Similarly, women were more likely to have suffered either a miscarriage (OR 1.59 (95% CI 1.42, 1.79)) or a stillbirth (OR 1.80 (95% CI (1.46, 2.21)) if they reported having experienced seizures. Women who reported hypertension were more likely to have had a skilled birth attendant (OR 1.16 (95% CI 1.08, 1.24)) and more likely to deliver at a health facility (OR 1.13 (95% CI 1.06, 1.21)). Similar associations of these variables were found for those women who reported having had seizures. (Table 4, Table 5)

**Table 4:** Predictors of self-reported hypertension in pregnancy.

Variables	Obstetric history of hypertension in the last 12 months (self-reported)		Crude OR (95% CI) Mean	Adjusted OR (95% CI)
	No	Yes	Mean difference (95% CI)	
	N (%)	N (%)		
	Mean ± SD	Mean ± SD		
Age	26.89 ± 4.36	27.81 ± 4.39	1.02 (1.01,1.03)	1.04 (1.04,1.05)
*Socio-economic status:*				
Poorest + poor	2845 (40.9%)	4785 (39.9%)	0.99 (0.93,1.06)	1.02 (0.95,1.10)
Middle	1249 (18.0%)	2353 (19.6%)	1.11 (1.02, 1.21)	1.12 (1.02,1.22)
Rich + richest	2862 (41.1%)	4842 (40.4%)	Reference	Reference

*Education level of female head of household*				
Less than class one	2668 (96.8%)	4531 (97.1%)	0.94 (0.82,1.08)	NA*
Class one or higher	87 (3.2%)	133 (2.9%)	Reference	

*Pregnancy outcome*				
Live birth	6024 (96.9%)	9693 (95.8%)	Reference	Reference
Stillbirth	191 (3.1%)	426 (4.2%)	1.38 (1.16,1.64)	1.34 (1.13,1.60)

*Type of birth attendant*				
Skilled birth attendant	4049 (64.9%)	6932 (68.2%)	1.16 (1.08,1.24)	1.28 (1.01,1.61)
Unskilled birth attendant	2191 (35.1%)	3230 (31.8%)	Reference	Reference

*Place of delivery*				
At health facility	3980 (63.8%)	6781 (66.7%)	1.13 (1.06,1.21)	1.07 (0.85,1.35)
Outside health facility	2260 (36.2%)	3381 (33.3%)	Reference	Reference

Delivery and related information is missing for n = 2725 (13.9%) women, for high blood pressure n = 648(3.3%) reported ‘Don’t know’, for seizures n = 316 (1.7%) reported ‘Don’t know’ and n = 193(1%) were missing.

* variable not included in final model for adjustment.

**Table 5:** Predictors of self-reported seizures in pregnancy.

Variables	Obstetric history of seizures in the last 12 months (self-reported)		Crude OR (95% CI) Mean difference (95% CI)	Adjusted OR (95% CI)
	No	Yes		
	N (%)	N (%)		
	Mean ± SD	Mean ± SD		
Age	27.41 ± 4.39	27.91 ± 4.55	1.02 (1.01,1.03)	1.02 (1.01,1.03)

*Socio-economic status*				
Poorest + poor	6819 (40.3%)	976 (42%)	1.20 (1.09,1.33)	1.27 (1.14,1.42)
Middle	3157 (18.6%)	523 (22.5%)	1.39 (1.24,1.57)	1.43 (1.25,1.63)
Rich + richest	6960 (41.1%)	826 (35.5%)	Reference	Reference

*Education level of female head of household*				
Less than class one	6456 (88%)	191 (89.7%)	0.92 (0.73,1.15)	NA*
Class one or higher	879 (12%)	22 (10.3%)	Reference	

*Pregnancy outcome*				
Live birth	14,206 (96.5%)	1772 (93.9%)	Reference	Reference
Stillbirth	512 (3.5%)	115 (6.1%)	1.80 (1.46,2.21)	1.71 (1.38,2.10)

*Type of birth attendant*				
Skilled birth attendant	9771 (66.1%)	1363 (72%)	1.31 (1.18,1.46)	1.95 (1.42,2.66)
Unskilled birth attendant	5011 (33.9%)	531 (28%)	Reference	Reference

*Place of delivery*				
At health facility	9591 (64.9%)	1318 (69.6%)	1.23 (1.11,1.37)	1.41 (1.04,1.92)
Outside health facility	5190 (35.1%)	576 (30.4%)	Reference	Reference

Delivery and related information is missing for n = 2725 (13.9%) women, for high blood pressure n = 648(3.3%) reported ‘Don’t know’, for seizures n = 316 (1.7%) reported ‘Don’t know’ and n = 193(1%) were missing.

* variable not included in final model for adjustment.

After adjustment for age, SES, skilled attendant at delivery and place of delivery, stillbirth remained significantly associated with self-reported hypertension in the most recent pregnancy (OR 1.36; 95% CI 1.14, 1.61). Having a skilled birth attendant and being in the middle SES class also appear to increase the likelihood of self-reporting pregnancy complications in this cohort, after adjustment (Table 4). Similarly, SES and type of birth attendant were significantly associated with reporting of seizures in pregnancy. Odds of having stillbirth were twice (AOR 1.71 (95% CI (1.38, 2.10)) in women who mentioned experiencing seizures compared to those who did not. These women were also highly likely to deliver at a health facility (AOR 1.41 (95% CI (1.04, 1.92)) (Table 5). The size of household, crude birth rate, proportion of live births, miscarriages and stillbirths and were all compared with national estimates (Table 6). Generally, our sample appears representative of the national population.

**Table 6:** Comparison of study area, provincial and national estimates of socio-demographics, pregnancy outcome and morbidity estimates.

Indicators	Household survey estimates Mean ± SD	Provincial estimates Mean ± SD	National estimates Mean ± SD
*Scio-demographic indicators*			
Size of household	6.7	6*	6.8
Houses headed by men	70%	Not available	89.1%
Improved drinking water	99%	93%*	93%
Improved sanitation	37%	49%*	59.5%
Flooring material:		Not available	
Mud or sand	67.1%		41.6%
Cement	30.2%		33.5%
Tiles	2.1%		1.1%

*Pregnancy outcomes and morbidities*			
	(N = )	(N = )	(N = )
Pregnant at time of survey	11.9%	8.7%	8%
	(N = )	(N = )	(N = )

*Place of delivery in last pregnancy*			
Home	34.2%	41.4%	51.6%
Government hospital	30.1%	14%	14.6%
Private hospital	35.2%	44.6%	33.6%
Last delivery by a skilled attendant	66.7%	60.5%	52.1%
Recent pregnancy outcome			
- live birth	83%	83%	83.5%
- stillbirth	3.5%	3%	2.8%
- miscarriage	13%	14%	13.7%
Severe headache in pregnancy	72%	58.2%	48%
Seizures in pregnancy	12%	5.3%	3.7%

All estimates taken from PDHS 2006–07 and 2012–13 unless stated otherwise

* Estimates taken from Report on the Status of Millennium Development Goals Sindh and Official website of Government of Sindh

## 5 Discussion

In the absence of robust health information and vital registration systems, these findings provide first hand community-derived population demographics, morbidity and mortality estimates in MWRA and children in Sindh, Pakistan. Some rates were consistent with reported provincial and national rates while others showed significant disparity.

Compared with provincial estimates, the study area had, on average, one more household member (6 vs 6.7). Recent PDHS reported that 89% of households had a male head while 70% of surveyed houses in this study were headed by men [15]. This difference in proportion of male heads of household may be due to migration of males to either urban centers or other areas with greater employment opportunities. Unfortunately, provincial estimates for this indicator are not available to support this assumption.

The proportion of the study population with access to improved drinking water was higher, and access to improved sanitation was lower, than the provincial and national rates. Access to piped water was 50% less than rates reported earlier by other sources [8]. According to UNICEF and PDHS, 19% of the rural population of Pakistan had access to piped water but in this survey only 8% reported access (6). Approximately half (43%) of all households did not have any toilet facility and were using open bush or field whereas national estimates are lower (31.5%) [15]. These inconsistencies reflect that either district- or smaller unit-level estimates may differ from aggregated large level rates and a priority should be given to collect data from a specific targeted area prior implementing any intervention.

Obstetric complications were reported to be unusually high compared to estimates reported in literature. Prevalence of severe headache in last pregnancy in this survey was 72% vs 58.2% reported in a country-wide survey [8]. During this survey, women were asked if they had ever experienced high blood pressure or seizures in a past pregnancy; rates were found to be 62.1% and 11.9% respectively. Similarly, the PDHS collected information regarding pregnancy complications; however, instead of high blood pressure PDHS survey asked about related symptoms (swelling of hands and face, blurred vision and epigastric pain). For any of these symptoms reported, the percentage was close to or less than 25% and for seizures its was 4% [8]. There is a dearth of community-based data for rates of hypertension or convulsions during pregnancy with which to compare our findings. A hospital-based study from Sindh found pregnancy induced hypertension in 37% of pregnant women [18].

Community-based studies from neighboring countries have reported low prevalence of pre-eclampsia (1.4% India; 6.8% Bangladesh) [19], [20]. The difference between our study and the relevant literature is most likely because these studies objectively measured the blood pressure of pregnant women in the community and did not rely on self-reporting. We conducted the study in rural communities which has poor access to health facilities hence we could not verify the reported hypertension against medical diagnosis. This is an important learning from our study that in countries like Pakistan where health literacy is low, estimation of disease burden should include objective measurement to confirm the reported diseases. Onur et al. found high disagreement between reported and objectively measured disease in India [21]. However they found underestimation through self-reporting which is opposite to our findings but it supports our findings that in LMICs self-reported disease estimates can be erroneous. Also the seizure itself and its etiology were also not confirmed in this survey. Epilepsy is prevalent in Pakistan with a rate of 9.99 per 1000 population [22]. It may partially explain the reasons for high rates of self-reported seizures as community members cannot differentiate between causes of seizures and it was not verified against clinical diagnosis.

Due to the high reported rates of hypertension and seizures in pregnancy, it was important to determine characteristics that may be associated with these high rates of reporting. Illness reporting can vary based on an individual’s understanding of the causes, presentation and consequences of the condition [23]. Women have been shown to report ill health more often than men [24]. This gender differential may help to explain the high rate of morbidities reported here. Other predictors of self-perceived poor health identified in literature were lower education and socio-economic status [25]. Results of this survey are consistent with previous research indicating factors associated with self-rated ill health. No studies focused on self-rated health during or after pregnancy and whether pregnancy can affect perceptions of health are not available for comparison. A related qualitative study demonstrated that headache, anxiety and anger are reported by women or men as hypertension in this same population [24] which shows lack of awareness in community regarding hypertension in general and in pregnancy.

**Fig. 2:**
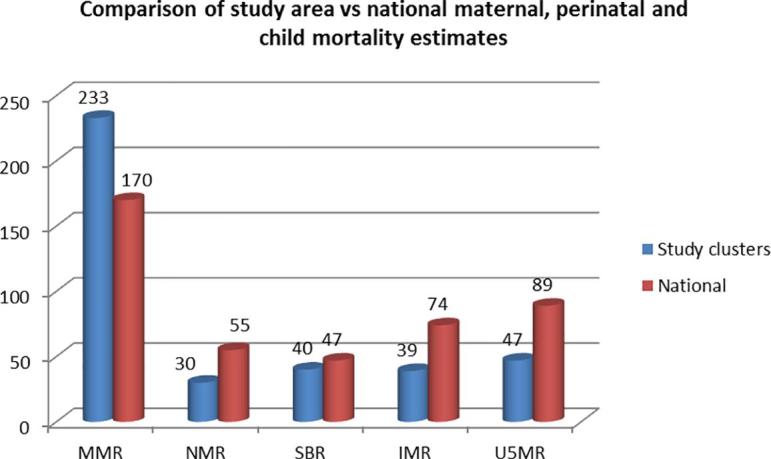
Comparison of study rates and national estimates for maternal, perinatal and child mortality.

Pakistan has a high birth rate (30.7/1000 population) [8], this survey was no different (CBR 28.4/1000 population). Neonatal, infant, and child mortality rates were 50% less than national estimates; and many of these deaths occurred at home. Half of the reported neonatal deaths and three quarters of deaths in children under five occurred at home. Literature from this part of world has reported negligence towards health care seeking for female children and a preference to preserve health of male children. However, in this study we cannot conclude if there was a differential rate associated with gender. (Fig. 2.)

The caveat of our study is that we could not validate the reported HTN and seizures through medical records for any of the participants. Hence, we caution readers to interpret the estimates of HTN and seizures in the context of study’s limitation. Except from these two estimates our study added valuable literature on community based estimates of maternal and neonatal health indicators from Pakistan, large sample size and first study to report the factors of self-reported maternal diseases.

## 6 Conclusion

Despite an established health information system there is a lack of quality vital statistics available in Sindh [5], [6]. The findings from this household-based survey provide a rich insight into the health of rural communities in Pakistan. When the results are observed through the lens of the preceding feasibility study [26], there is a greater understanding of some aspects of women’s reproductive behavior, their perception of the pregnancy complications experienced by them and the community’s health-seeking behavior. This information will be invaluable for the research community to plan interventions for maternal and child health and estimates of community-level disease rates.

It is recommended that the government concentrate its efforts to monitor the data collection processes and validate data through community surveys of representative sampled households. In addition, it is important to disseminate these data to other partners and stakeholders who can help the government design strategies for improved health outcomes and increased community awareness of the life-threatening complications of pregnancy.

## 7 Declarations

### 7.1 Ethics approval and consent to participate

This study received ethical approval from Ethics Review Committee of Aga Khan University, Karachi, Pakistan; (ERC#1917-Obs-ERC-11); National Bioethics Committee of Pakistan (4–87/NBC-104/12/RDC/1895) and the Institutional Review Board of University of British Columbia, Vancouver, Canada. Participants provided independent written consent to participate in the study. For the participants younger than 19 years of age legal guardian i.e. husband of the women or in-laws gave the consent.

## 8 Consent for publication

Not Applicable.

## 9 Availability of data and materials

The datasets used and/or analyzed during the current study are available from the corresponding author on reasonable request.

## Funding

This study was funded by the University of British Columbia PRE-EMPT (Pre-eclampsia/Eclampsia, Monitoring, Prevention and Treatment) initiative supported by the Bill & Melinda Gates Foundation. Granting agency has no role in designing of the study and collection, analysis, and interpretation of data and in writing the manuscript.

## Authors’ contributions

SS was involved in conceptualization, designing, execution of the study, analyzing the data, writing and reviewing the manuscript. RNQ was involved in the concept, designing and supervision of the execution of the study. She also contributed in writing and review of the manuscript; FR, JM were involved in the design and execution of the study; data collection. IA, TL were involved in data curation, MV and BAP contributed to the writing of the manuscript. DS, ZAB and PvD made significant intellectual contribution to the entire study. All authors read and approved the final manuscript.

## Declaration of Competing Interest

Authors declare no competing interests.
